# A grumbling concern: an international survey of gastrointestinal symptoms in cystic fibrosis in the modulator era

**DOI:** 10.3310/nihropenres.13384.1

**Published:** 2023-04-14

**Authors:** Rebecca J Calthorpe, Natalie Goodchild, Vigilius Gleetus, Vinishaa Premakumar, Bu Hayee, Zoe Elliott, Bethinn Evans, Nicola J Rowbotham, Siobhán B Carr, Helen Barr, Alexander Horsley, Daniel Peckham, Alan R Smyth

**Affiliations:** 1 University of Nottingham School of Medicine & NIHR Nottingham Biomedical Research Centre, Nottingham, UK; 2 Patient and Public representative, N/A, UK; 3 Kings College Hospital NHS Foundation Trust, London, UK; 4 Royal Brompton Hospital (part of GSTT) and Imperial College, London, UK; 5 Nottingham University Hospitals NHS Trust, Nottingham, UK; 6 University of Manchester & NIHR Manchester Biomedical Research Centre, Manchester, UK; 7 Leeds Institute of Medical Research at St James’s, Leeds, UK

**Keywords:** Respiratory, cystic fibrosis, gastrointestinal symptoms, CFTR modulators

## Abstract

**Background:**

Gastrointestinal symptoms in cystic fibrosis (CF) are common and intrusive to daily life. Relieving gastrointestinal symptoms was identified as an important research priority and previously explored in an international survey in 2018. However, following the widespread introduction of cystic fibrosis transmembrane conductance regulator (CFTR) modulators in 2019, the landscape of CF treatment has changed. We repeated an online survey to further describe gastrointestinal symptoms and their effect on quality of life (QoL) in the CFTR modulator era.

**Methods:**

An electronic survey consisting of closed questions and free text responses was distributed via social media and professional networks for a period of one month between March - April 2022. People with CF (pwCF), their family and friends, and healthcare professionals (HCPs) were invited to take part.

**Results:**

There were 164 respondents: 88 pwCF (54%), 22 (13%) family, and 54 (33%) healthcare professionals (HCPs). A total of 89/110 (81%) pwCF or family members reported CFTR modulator treatment. The most commonly reported symptoms were wind / gas, rumbling stomach noises, loose motions (modulator) and bloating (no modulator). Abdominal pain and bloating had the greatest impact on QoL.

For those on a CFTR modulator, the proportion of pwCF reporting “no change” or “worse” for all of the symptoms surveyed was greater than the proportion reporting an improvement. Following modulator introduction, dietary changes were recommended by 28/35 (80%) of HCPs and reported by 38/76 (50%) lay respondents. Changes in medication were recommended by 19/35 (54%) HCPs and reported by 44/76 (58%) of patients and family members.

**Conclusion:**

This survey has shown that gastrointestinal symptoms remain prevalent in pwCF in the CFTR modulator era, though the nature of these symptoms may have changed. A better understanding of the underlying pathophysiology of these symptoms is essential. Future clinical studies should focus on improving symptoms and QoL.

## Plain language summary

What is already known: Gastrointestinal symptoms are common and intrusive to everyday life for people with cystic fibrosis (CF), however the majority of studies reporting gastrointestinal symptoms in CF are published prior to the widespread introduction of cystic fibrosis transmembrane conductance regulator (CFTR) modulator therapies. These are medications which target the underlying defect in CF rather than the consequences of CFTR failure.

What this study adds: Through this survey, we describe the similarities and differences of gastrointestinal symptoms for people with CF on modulator therapy compared to those not receiving modulators. Comparisons were also made to our previous work which was completed in 2018 prior to the licencing of the newest, and most widely used modulator, Elexacaftor / Tezacaftor / Ivacaftor (ETI).

How this study might affect future research: This survey provides a snapshot into gastrointestinal symptoms for people with CF which will be of benefit for researchers as well as clinicians caring for people with CF. These results will inform the development of a CF-specific gastrointestinal patient reported outcome measure for people with CF that can be used in clinical trials.

## Introduction

Cystic fibrosis (CF) is an autosomal recessive, life-limiting condition affecting approximately 100,000 people worldwide, caused by mutations to the gene encoding the cystic fibrosis transmembrane conductance regulator (CFTR) protein ^1^ [ Cystic Fibrosis FAQs - What is cystic fibrosis?]. It is a chronic multi-system disorder with the gastrointestinal tract being an important cause of morbidity for people with CF (pwCF). Common gastrointestinal symptoms include abdominal pain, flatulence, bloating, and foul-smelling stools ^2, 3^, with over one in five pwCF reporting moderate to severe gastrointestinal symptoms ^4^. Approximately 85% of pwCF are pancreatic insufficient necessitating the need for pancreatic enzyme replacement therapy (PERT) and between 2–5% each year will develop distal intestinal obstruction syndrome (DIOS) ^5, 6^.

One of the most important research priorities identified by the CF community in the first James Lind Alliance Priority Setting Partnership (JLA PSP), published in 2018, was ‘How can we relieve gastrointestinal symptoms such as stomach pain, bloating and nausea?’ ^7^ and also remained a priority for research in the recent JLA PSP refresh of priorities in 2022, described below [ Cystic Fibrosis Refresh Top 10 priorities]. This research question was further explored in 2018 using an international online survey involving pwCF, their family and friends, and healthcare professionals (HCPs) ^3^. The survey identified the high prevalence of gastrointestinal symptoms in pwCF and negative impact on quality of life, with two thirds of respondents reporting missing school or work due to significant gastrointestinal symptoms ^3^. At this time, modulator therapy was only licenced and available for a minority of pwCF.

More recently, the widespread introduction of the CFTR modulator combination of Elexacaftor / Tezacaftor / Ivacaftor (ETI) (Kaftrio® / Trikafta®, Vertex Pharmaceuticals) in 2019 has changed the landscape of CF treatment. ETI has led to dramatic improvements in respiratory health for patients, including improvements in lung function, reduced pulmonary exacerbations and improved CFQ-R respiratory domain scores, indicating improved quality of life ^1, 8– 10^. The impact of ETI on the gastrointestinal tract is less well characterised. Early reports suggest some improvement in gastrointestinal symptoms after initiation of ETI therapy ^11^. This was demonstrated in a prospective study of gastrointestinal symptoms with modest improvements in symptoms at 24 weeks compared to baseline using the CF-specific CFAbd-Score ^11^. Similar small improvements were reported in the PROMISE study (change of scores at 6 months compared to baseline, PAGI-SYM -0.15, PAC-SYM -0.14, PAC-QOL -0.15) ^12^. In the first study by Mainz *et al.*, no sex differences were noted in the reporting of GI symptoms although the PROMISE study demonstrated high scores at baseline within female participants.

Additionally, earlier studies which evaluated the effects of Ivacaftor on those with a gating mutation also demonstrated improvements to the proximal small intestinal pH ^13^, changes in the gut microbiome and decreased intestinal inflammation ^14^. Conversely, in a phase 3 randomised control trial, diarrhoea was reported as one of the most common adverse events in patients on ETI compared to placebo (12.9% vs 7% respectively) ^1^ and was one of the 15 most commonly reported adverse symptoms identified in a systematic review of the four available CFTR modulators currently in clinical practice ^15^. In a recent JLA refresh into research, relieving gastrointestinal symptoms remained a key research priority and additionally, “what are the effects of modulators on systems outside the lungs such as pancreatic function, liver disease, gastrointestinal, bone density etc” was identified as a new top 10 priority in the CFTR modulator era [ Cystic Fibrosis Refresh Top 10 priorities]. This indicates that gastrointestinal symptoms continue to be a problem for some pwCF despite widespread commencement on ETI therapy.

The aim of this international survey was to further explore gastrointestinal symptoms in pwCF and the impact of CFTR modulators on these and associated quality of life. These results will also contribute to the development of a CF-specific patient reported outcome measure (PROM) that aims to capture the daily burden of gastrointestinal symptoms for pwCF (visit cftummytracker.org for more information). Having a current knowledge of the landscape of gastrointestinal symptoms is essential in order for this PROM to be relevant to its intended population group ( clinicaltials.gov NCT05251467). Preliminary results of this survey were published as a conference abstract from the 2022 North American Cystic Fibrosis Conference (NACFC) ^16^.

## Methods

### Patient and public involvement

Patients and the public (as well as health professionals) took part both in the JLA PSP and in the recent refresh exercise, both of which have identified gastrointestinal problems in CF as a priority question for clinical research. People with CF and parents of children with CF were members of the study steering group. A person with CF is a co-author on this paper and helped to design the questionnaire, publicise the project via social media and interpret the qualitative data. They have contributed to writing the manuscript and disseminating the findings.

### Survey development

This work was led by a steering group representative of the CF community, consisting of adults and children with CF, parents of pwCF and multidisciplinary HCPs and researchers who are part of a wider research study: a Comprehensive Approach to Relief of Digestive Symptoms in Cystic Fibrosis: CARDS-CF ( NCT05251467). Researchers were healthcare professionals specialising in adult and paediatric respiratory medicine, cystic fibrosis and gastroenterology. In addition, some members of the research team were instrumental in the development of both JLA PSPs in CF, involved in the analysis of the original gastrointestinal symptom survey in CF or completed similar research in the exploration of other priority research questions in which the same methodology was used ^17^. Researchers used their own social media accounts to promote the survey but had no direct contact with participants.

The present survey aimed to gather quantitative and supporting qualitative data on gastrointestinal symptoms in the CFTR modulator era. Approximately 90% of pwCF have a mutation eligible for treatment with ETI [ CF Trust - Fighting for life-saving drugs], although funding arrangements vary from country to country and the drug is not universally available. The survey for this study was developed by the steering group described above and questions were also drawn from the original 2018 survey ^3^ (see participant information sheet and 2022 survey ^18, 19^). This was to allow comparison of results, where appropriate. Members of the patient community co-designed the survey to ensure the most relevant and appropriate questions were used and that the wording was clear. Ethical approval was given by the University of Nottingham Research Ethics Committee (REC) (Ref: FMHS 436-0122, approved 11/02/2022).

An electronic questionnaire was generated using SurveyMonkey.com. Participants were shown an introductory page containing a description of the survey and a weblink to a more detailed participant information sheet including information on how their data would be collected and used, General Data Protection Regulation (GDPR) information and a link to the University of Nottingham privacy policy ^19^. Participants were asked to read and give consent prior to taking part. Those under the age of 16 years were advised to get permission from their parents or guardians. Questions were divided into those for HCPs and pwCF (which were further sub-divided by modulator status). The survey consisted of a series of yes/no questions, multiple-choice questions, Likert scales and free text responses and used skip logic to allow participants to navigate to the most appropriate question based on their responses.

Participants were asked questions which were developed around the following themes:

◦ Presence of gastrointestinal symptoms for pwCF and their effect on quality of life
◦ Effect of CFTR modulators on gastrointestinal symptoms and quality of life (where appropriate)
◦ Dietary or medication changes to manage gastrointestinal symptoms

### Data collection

The survey was open for one month between March and April 2022 and was promoted through social media platforms such as Twitter using the Twitter handles @CFAware, @QuestionCF, @CARDSCFresearch and professional accounts, as well as on Instagram and Facebook. In addition, the survey was promoted to health professionals via professional organisations such as the UK CF Medical Association. In order to gain the experiences of as many people as possible, the survey was open to all pwCF, their friends and family and HCPs caring for pwCF. There was no pre-determined target sample size. The survey was anonymous although participants were given the option of leaving their contact details in order to receive the results or be involved in any future research opportunities relating to the survey. Participants were made aware that these would be separated from their survey results to maintain anonymity. In addition to questions relating to a person’s experience of gastrointestinal symptoms in CF, participants were asked to self-report on basic demographic information such as country they lived in, age, and gender (recorded as “male”, “female”, “prefer not to say” and “other” with the free text option to self-identify if they wished).

### Data analysis

Data were downloaded into Microsoft Excel and participant responses were separated from their contact details prior to analysis and stored as per GDPR guidelines. Analysis was informed by an analytical approach which was previously developed and used by the group through a combination of descriptive statistics, qualitative content analysis and thematic analysis, where appropriate ^17, 20^. Closed responses were analysed using Microsoft Excel and descriptive statistics were used for interpretation. Data generated from pwCF and HCPs were reviewed separately and responses from pwCF were separated by modulator status. Where questions for this survey were also included in the 2018 symptom survey, the raw data for each data set were described and compared.

Questions which offered an additional free text response were downloaded into NVivo 12 package (QSR International, Massachusetts) for thematic analysis in order to help support overall understanding of the question. The free text responses for each question were initially reviewed to identify possible themes within the responses. The word frequency function was used to aid with this. Related words (such as bloat, bloated, bloating) were combined whilst other words which were felt to be either artificially increased as they were included within the question (for example diet or medication) or did not relate to the results (verbs such as get, made), were removed.

Through this review of the free text responses, we identified overarching areas of interest in the data (termed themes) and more specific areas of interest within this (termed codes). All the free text responses for the survey were then reviewed and mapped to these codes. Given the variation in the length of free text responses submitted, some responses were relevant to more than one code, therefore these data could be mapped to multiple codes or themes as appropriate. As well as the identification of key themes in the results, alternative or more minority opinions were also considered. The coding and analysis of free text responses were performed independently by two authors and checked by a third researcher in order to ensure consistency and appropriateness of how the data were assigned to each code or theme. 

## Results

A total of 167 people consented to take part in the survey, with 164 people completing some aspect of the survey, comprising 88 pwCF (54%), 22 (13%) parents or other family members and 54 (33%) HCPs. The median age of pwCF (as self-reported or reported by a family member) was 33 years (range 3 – 62 years), female participants 90/127, (71%), male participants 37/127 (29%). We received responses from 11 countries although the majority of responses received were from the UK (107/126, 85%). There was a greater proportion of responses from UK patients than in the 2018 survey (previously 171/276, 62%). Dietitians accounted for almost half of the responses from HCPs (24/54, 44%). Not all participants answered every question and so the denominator has been included where response numbers and percentages are given. The respondent demographics are in Table 1.

**Table 1.** Demographic information.

Demographics	n (%)
**Population group (n=164)**	
People with CF	88 (54%)
Parents or other relative	22 (13%)
HCP	54 (33%)
- Dietitian - Respiratory physician - Doctor: other - Nurse - Other - Unknown	- 24 (44%) - 13 (24%) - 6 (11%) - 6 (11%) - < 5 - < 5
**Gender (n=127)**	
Female	90 (71%)
Male	37 (29%)
**Country (n = 126)**	
United Kingdom USA and Canada Europe (excluding UK) Rest of the world	107 (85%) 11 (9%) 7 (6%) < 5

**Figure 1.**
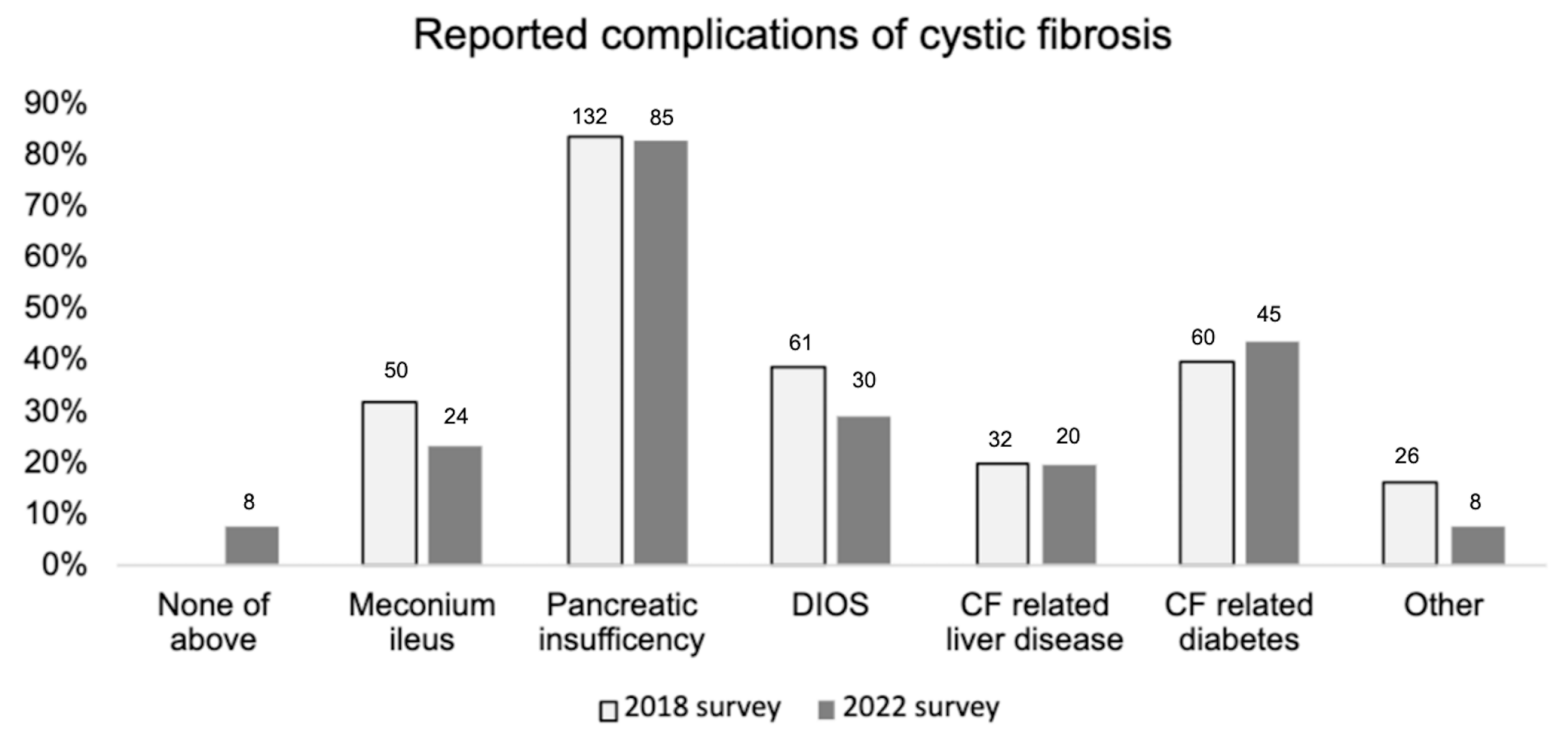
Reported history of CF related complications in the 2018 and 2022 surveys. 2018 survey n=157, 2022 n=103 responses. “None of above” response option not given as part of 2018 survey.

For pwCF, 89/110 (81%) were prescribed a CFTR modulator. ETI was the modulator most commonly reported (73/84, 87%). Most reported starting in 2020, corresponding with the UK-wide funding of ETI through the National Health Service (NHS). Of those pwCF not prescribed a CFTR modulator (n=20), four had their modulator discontinued due to adverse effects, including gastrointestinal adverse effects. Reported gastrointestinal complications were comparable between the 2018 and 2022 surveys ( Figure 1).

## Symptoms experienced

those participants not taking a modulator, 17/19 (89%) reported experiencing gastrointestinal symptoms. For those who were commenced on a CFTR-modulator, 58/84 (69%) reported symptoms prior to commencing therapy and 60/84 (71%) after initiating treatment. The vast majority of HCPs (51/52 98%) said that they cared for patients with gastrointestinal symptoms.

**Figure 2.**
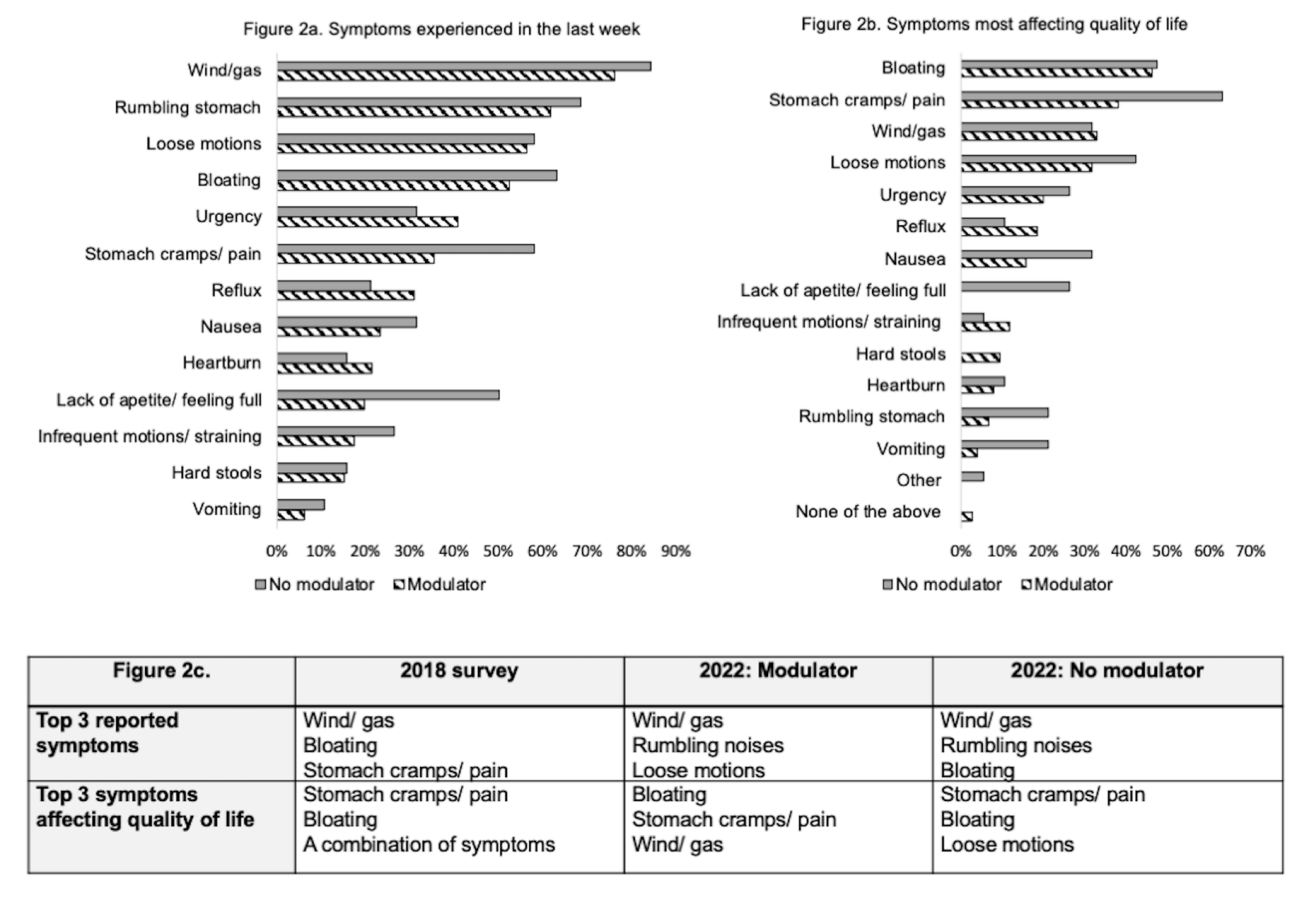
**2a**. Symptoms experienced by pwCF at least once a week by modulator status. **2b**. Symptoms most affecting quality of life by modulator status. **2c**. Top 3 symptoms reported with comparison to 2018 survey.

Figure 2a shows the frequency of gastrointestinal symptoms that are experienced at least weekly for pwCF, separated by modulator status. The most commonly reported symptoms for both groups were: wind/gas, rumbling stomach noises, loose motions (modulator) and bloating (non-modulator). For the majority of symptoms, a greater proportion of patients who were not on modulator therapy reported each symptom.

Comparisons between the top 3 reported symptoms by pwCF in the 2018 survey and this survey show that stomach pain and bloating were in the top 3 symptoms for both surveys ( Figure 2c). Direct comparison of the question was not possible as some response options which were combined in 2018 were separated in this survey. For example, loose/frequent bowel motions were separated into two response options, whilst other were not included, such as “a combination of symptoms”. Symptoms most commonly reported to HCPs by those not on modulators were constipation (25/38 66%), bloating (21/38 55%) and stomach pain (18/38 47%).

**Figure 3.**
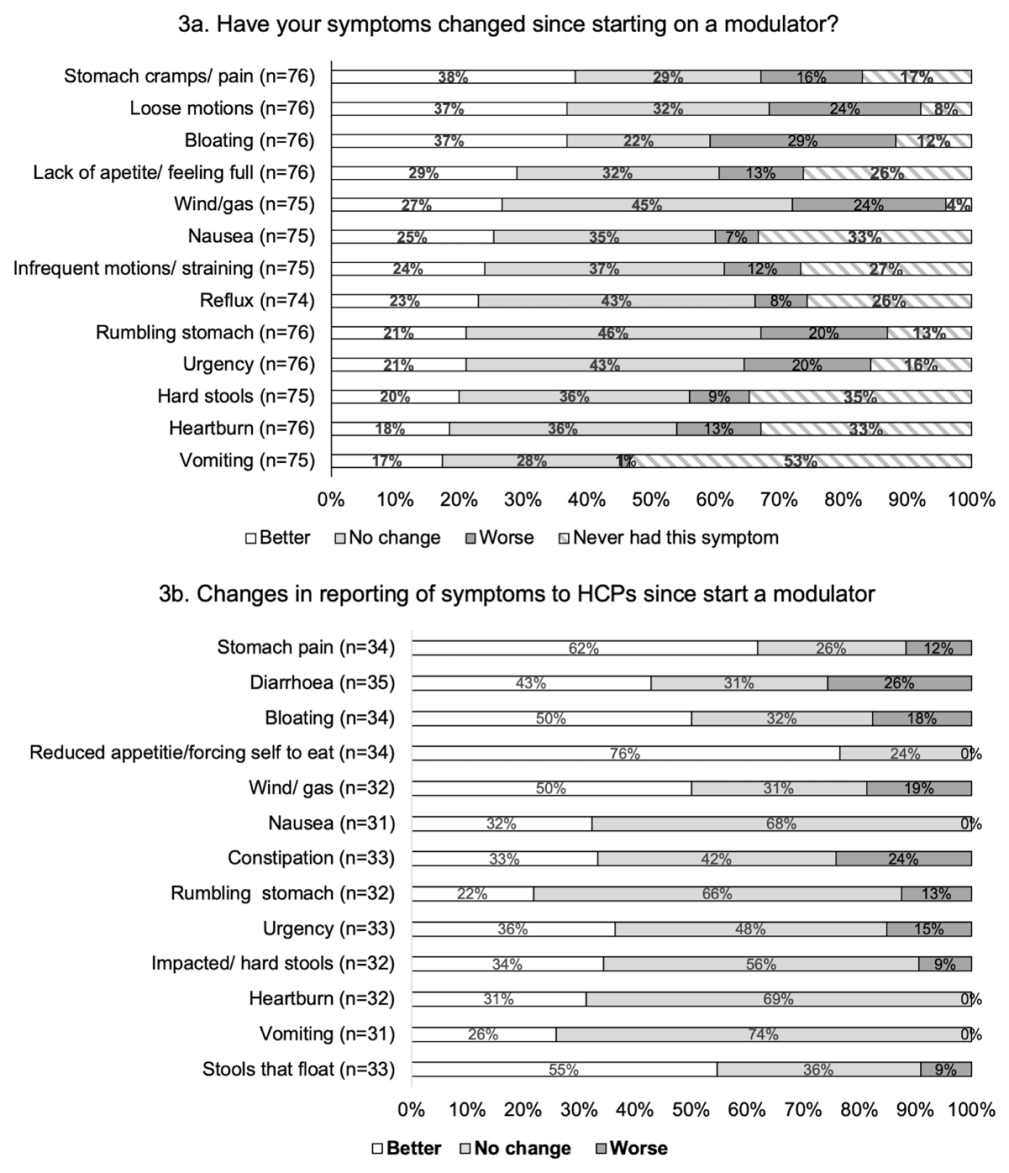
Changes to gastrointestinal symptoms experienced since starting on a CFTR modulator. **3a**: PwCF were asked to compare how their symptoms had been in the last 4 weeks, compared to prior to initiating modulator therapy. **3b**: Reporting of gastrointestinal symptoms to HCPs in those people taking a CFTR modulator.

Those pwCF on CFTR-modulator therapy and HCPs were asked whether they felt gastrointestinal symptoms had improved, stayed the same or worsened since initiating CFTR modulator treatment. PwCF were asked to consider their gastrointestinal symptoms over the previous 4-week period ( Figure 3a). For each of the 13 symptom categories, the proportion of pwCF reporting “no change” or “worse” symptoms, following the start of CFTR modulator therapy, was greater than the proportion reporting an improvement.

HCPs shared similar experiences where the greatest proportion of respondents either reported “no change” or “worse” symptoms for most of the symptom categories ( Figure 3b). The exceptions to this were reduced appetite, stomach pain and stools that float where the greatest proportion of responses reported an improvement in these symptoms.

In free text responses, HCPs reflected on the variable nature of gastrointestinal symptoms in response to modulators e.g.

*HCP quote 1: “symptoms highly variable, for some people things improve and for others they worsen!”*

## Quality of life

Both groups were asked to what extent they agreed with the statement “gut symptoms affected the QoL for pwCF”, with responses on a 5-point Likert scale. Overall, 84/95 (88%) pwCF and 33/35 (94%) HCP said they agreed or strongly agreed with this statement.

Figure 2b and 2c shows the most common symptoms affecting quality of life for pwCF, with comparisons of the top 3 symptoms with the 2018 data. Pain and bloating remained the symptoms felt to most impact quality of life and this opinion was also shared amongst HCPs. HCPs identified the top symptoms most affecting quality of life for pwCF to be stomach pain (22/35, 63%), constipation (16/35, 46%) and bloating (15/35, 43%).

**Figure 4.**
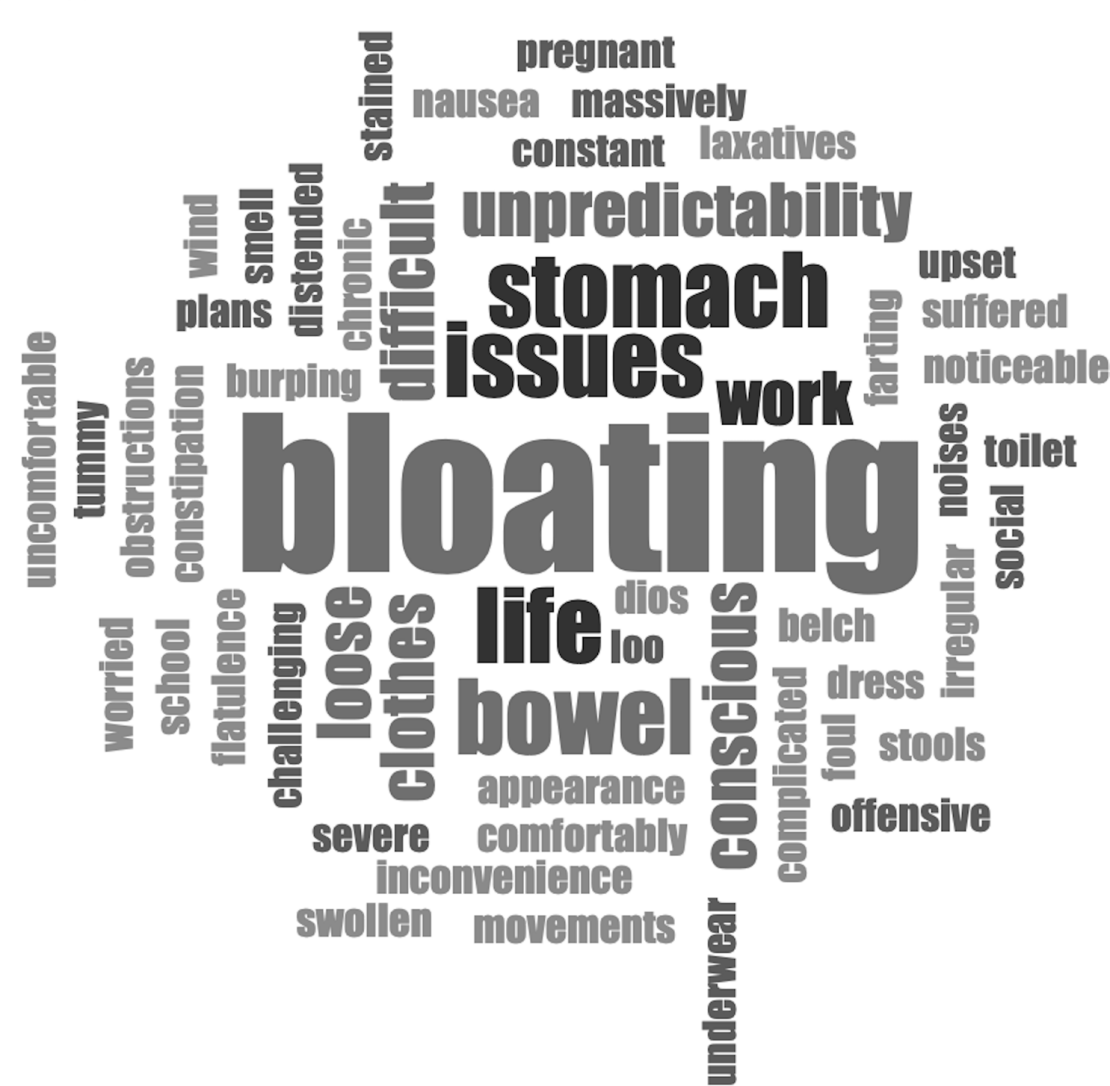
Word cloud describing feelings of embarrassment caused by gastrointestinal symptoms for pwCF on modulators.

Almost two thirds (62/100, 62%) of pwCF felt their gastrointestinal symptoms made them feel embarrassed or affected their self-confidence, although this was experienced to a greater extent in those not receiving CFTR-modulator therapy (modulator: 48/81 (57%) vs no modulator: 14/19 (74%)). These results were very similar to those reported in 2018 (94/145 65%). The theme of embarrassment was further explored through the free text responses ( Figure 4).

Bloating was the most commonly reported symptom in the free text responses causing embarrassment for people. Some respondents reported needing different clothes to conceal their bloating. This was also reported in the previous survey before ETI became available ^2^. 

PwCF quote 2: *“When I started Kaftrio I suddenly began to get massively bloated. I looked heavily pregnant and it was a very noticeable change to my body. It affected how I felt about myself both because I was heavily bloated and because it was difficult to dress comfortably or have clothes fit properly.”*

PwCF quote 3 *: “I’m embarrassed by my gut symptoms with wind, bloating, going to the bathroom multiple times. I get anxiety going to people’s homes in case I need to use the facilities and my gut is acting poorly.”*

Others talked about the impact of the gastrointestinal symptoms on social situations for example using the toilet in social situations, feeling worried about going out, or the unpredictability of symptoms. In 2018 two thirds of respondents missed school or work because of their gastrointestinal symptoms (97/146). In this survey, this was reduced to 31% (29/94), although this was higher in the non-modulator cohort (9/19 47% vs 20/75 27%). However, 43/94 (46%) of pwCF said they missed social occasions because of their gastrointestinal symptoms (modulator: 34/75 45% vs no modulator: 9/19 47%).

## Diet and medication changes

For those pwCF on modulators 44/76 (58%) reported having made changes to their medications which was similar to that reported by HCPs (19/35 54%). Half of pwCF (38/76, 50%) had made changes to their diet to manage gastrointestinal symptoms. This was lower than the proportion of HCPs (28/35, 80%) who reported making changes to the dietary advice they gave for gastrointestinal symptom management. 

Dietary changes made by pwCF were focused around two main themes. 1) Reducing the amounts of certain food groups such as carbohydrates, dairy and trialling an increased plant-based diet, and 2) maintaining a healthy diet through the reduction of fats and calorie intake to counteract the increased weight gain experienced on starting a modulator. A healthy diet was also promoted by HCPs following CFTR modulator initiation, who in addition to advising on reducing calories and fats, also promoted the use of “healthy fats” and one HCP also reported promoting exercise to help with weight loss.

For pwCF taking a CFTR modulator and HCPs, the symptoms of constipation and impacted stools were felt to have not changed overall in the multiple-choice responses described above (Figure 3). Dietary advice given in the free text responses to treat or prevent constipation included increasing fluids and fibre intake with a small number of HCPs having also discussed the use of laxatives for the management of this.

Common medication changes reported by pwCF after starting a modulator included the introduction or increasing the dose of proton pump inhibitors, in particular omeprazole for acid reflux (omeprazole word frequency, 7 times), and increased use of laxatives for the management of constipation. The word laxative and its synonyms were used 7 times in the free text responses for this question. Conversely, one person reported being able to stop laxatives since starting modulator therapy.

For HCPs the most discussed medication relating to this question was PERT, with PERT and its synonyms used 10 times. The two main themes surrounding PERT use were:

1) Changes made by HCPs to PERT doses: for example, reviewing, altering, reducing or stopping PERT

HCP, quote 4: *“We have managed to reduce or stop Creon in some cases but not all.”*

2) Changes made to PERT doses directly by their patients without the advice of HCPs. Reasons given for this included patient’s experiences of gastrointestinal symptoms, the perceived need for PERT had changed, or to counteract the weight gain seen following the introduction of modulator therapy.

## Discussion

This survey confirms that pwCF frequently experience gastrointestinal symptoms with the most common symptoms being similar to those described in our 2018 survey ^3^. These symptoms can affect quality of life for pwCF through disrupting school, work and social events and lead to feelings of embarrassment or self-consciousness. Although for some people gastrointestinal symptoms have improved, most noticeably for symptoms of pain, bloating and loose motions, overall, the proportion of respondents reporting “no change” or “worse” symptoms in each category of this survey, was greater than the proportion reporting an improvement after starting modulators.

Recent results from the PROMISE study, a prospective observational study of pwCF taking ETI demonstrated a small but statistically significant improvement in gastrointestinal symptoms which was felt unlikely to translate into a clinically meaningful benefit for patients ^12^. In contrast, in a prospective study of gastrointestinal symptoms, following the introduction of ETI, using a CF-specific questionnaire (CFAbd-Score) ^11^, Mainz *et al.* demonstrated an improvement in gastrointestinal symptoms. Improvements were most evident for abdominal pain intensity (20% improvement in abdominal pain intensity scores and 13% improvement in abdominal pain experienced scores). Bloating was also reduced by 12% ^11^.

It was encouraging to see in this survey that the percentage of pwCF missing school or work because of gastrointestinal symptoms had decreased compared to previously, although this was to a greater extent for those on modulators. This may also reflect the improvement in some gastrointestinal symptoms in this group. Unfortunately, the embarrassment experienced as a result of gastrointestinal symptoms showed little change compared to the results of the 2018 survey. Interestingly, embarrassment was increased in the study by Mainz *et al.* at 24 weeks following ETI initiation ^11^. They attributed this to a higher expectation of participants following a clinical improvement on therapy ^11^.

The majority of HCPs reported that following commencing of modulator therapy, they had altered their medication prescribing practices as well as dietary advice, in order to manage gastrointestinal symptoms. In some cases, HCPs were able to adjust a patient’s PERT, including reducing or stopping the medication. However, in other cases the patients were instigating changes to PERT prior to health care advice. PERT was previously identified as one of the most burdensome treatments for pwCF ^21^.

## Limitations

This study provides a snapshot of the occurrence of gastrointestinal symptoms in pwCF but inevitably the information is reliant on participant recall. We acknowledge that many pwCF were commenced on modulators in 2020, indicating a long recall time to the pre-treatment period (over a year). This could lead to recall bias. Similarly, it is possible that those individuals who have gastrointestinal symptoms which are particularly troublesome are more likely to respond to the survey compared to those where symptoms were not an issue. This could be reflected also in the proportion reporting CF complications such as meconium ileus (a risk factor for DIOS) which was higher in our survey (24/103, 23%) than in the UK CF registry (19%) ^6^. Nevertheless, the prevalence of pancreatic insufficiency was similar to that of the UK CF registry (2022 survey: 85/103 83%, UK CF registry 85%) ^6^. Additionally, although comparisons were drawn between the 2018 and 2022 data, we acknowledge that in order to maximise engagement and completion of the survey, the study populations were not standardised. Furthermore, although this survey was open to all pwCF, there was a greater response by females compared to male participants (females: 90/127, 71% vs males: 37/127, 29%). This may reflect the findings of recently published studies in CF that GI symptom scores were found to be higher in female participants ^4, 12^. However, we do acknowledge that sex differences were not seen in all studies, with studies by Mainz *et al.* finding no difference in GI symptoms based on sex ^2, 11^. The lower number of male participants prevented sub-analysis of the results by modulator status and gender and so conclusions around GI symptoms based on gender cannot be drawn in this survey.

In the present study, the number of individuals not on CFTR modulators (19%) was higher than expected, as approximately 90% of the CF population should be eligible for this treatment [ CF Trust - Fighting for life-saving drugs]. In addition to eligibility based on genotype, people may also have not had access to modulators due to the lack of funding in their healthcare system or they may have had modulator treatment discontinued, due to adverse effects. Finally, this survey was promoted and disseminated online and so its availability was limited to those who had access to digital technology. This may have limited those who choose not to engage with social media, lack internet access or a digital device and those from low- and middle-income countries from being able to give their experiences in the survey ^22^.

## Conclusion

This survey highlights that gastrointestinal symptoms still remain prevalent in the CFTR modulator era in pwCF. A better understanding of the underlying pathophysiology of these symptoms is essential in order to improve gastrointestinal symptoms for pwCF. Future clinical studies into gastrointestinal symptoms should focus on understanding and improving both the symptomatology and quality of life for pwCF.

## Data Availability

Ethical approval was granted by the University of Nottingham Research Ethics Committee. The approved patient information sheet detail that data will be stored within the University of Nottingham and that no participant with be personally identifiable from the results. This is also detailed in the approved data management plan and therefore the raw data has not been made publicly available. A redacted version of the data can be obtained by reasonable request to the study Principal Investigator and corresponding author Professor Alan Smyth ( alan.smyth@nottingham.ac.uk). This will be assessed on a case-by-case basis. Applications should state the research question being addressed and include a link to the researcher’s published protocol. This will be reviewed by the research team and a final decision to share data the responsibility of the Principal Investigator. figshare: Participant information sheet for online survey "The use of CFTR modulators and gut symptoms in Cystic Fibrosis. https://doi.org/10.6084/m9.figshare.22263952.v1 ^18^ figshare: 2022 GI symptom survey in cystic fibrosis.pdf. https://doi.org/10.6084/m9.figshare.22263886.v1 ^19^ Data are available under the terms of the Creative Commons Attribution 4.0 International license (CC-BY 4.0).
