# Supplementary material for: A grumbling concern: an international survey of gastrointestinal symptoms in cystic fibrosis in the modulator era
Source: NIHR Open Res. 2023 Apr 14;3:18. [Version 1] doi: 10.3310/nihropenres.13384.1 (PMC10593346; doi:10.3310/nihropenres.13384.1)
Supplement: Peer review history

# Reviewer report: Reviewer response for version 1

**DOI:** 10.3310/nihropenres.14515.r29560

**Published:** 2023-09-01

**Authors:** BARBARA RUARO

**Affiliations:** 1 University Hospital of Cattinara, Trieste, Italy

This is an interesting paper. The manuscript is quite well written. I have few suggestion:

 A) 1- Introduction

 " *Cystic fibrosis (CF) is an autosomal recessive, life-limiting condition affecting approximately 100,000 people worldwide, caused by mutations to the gene encoding the cystic fibrosis transmembrane conductance regulator (CFTR) protein ^1^* "

 Please improve this paragraph and some references. Please, add some information regarding the different aspects of *Cystic fibrosis.* I suggest:

1. Combined use of rheology and portable low-field NMR in cystic fibrosis patients. *Respir Med*. 2021;189:106623. doi:10.1016/j.rmed.2021.106623
2. Prevalence and Impact of Rheumatologic Pain in Cystic Fibrosis Adult Patients. *Front Med (Lausanne)*. 2022;8:804892. Published 2022 Feb 8. doi:10.3389/fmed.2021.804892

B) Please, add some information regarding the statistical analyses. The Authors need to add some information on statistical tests used to analysed the data and better clarify the value of the results

 C) Discussion

 " *This survey confirms that pwCF frequently experience gastrointestinal symptoms with the most common symptoms being similar to those described in our 2018 survey ^3^. These symptoms can affect quality of life for pwCF through disrupting school, work and social events and lead to feelings of embarrassment or self-consciousness. Although for some people gastrointestinal symptoms have improved, most noticeably for symptoms of pain, bloating and loose motions, overall, the proportion of respondents reporting “no change” or “worse” symptoms in each category of this survey, was greater than the proportion reporting an improvement after starting modulators.*"

 Improve the summary of the most important study results. Please, add some information regarding the *most important statistically significant  study results*

 D) Conclusion

 " *This survey highlights that gastrointestinal symptoms still remain prevalent in the CFTR modulator era in pwCF. A better understanding of the underlying pathophysiology of these symptoms is essential in order to improve gastrointestinal symptoms for pwCF. Future clinical studies into gastrointestinal symptoms should focus on understanding and improving both the symptomatology and quality of life for pwCF.*"

 Underline the novelty of the study and the possible clinical implications.

Is the work clearly and accurately presented and does it cite the current literature?

Yes

If applicable, is the statistical analysis and its interpretation appropriate?

Yes

Are all the source data underlying the results available to ensure full reproducibility?

Yes

Is the study design appropriate and is the work technically sound?

Yes

Are the conclusions drawn adequately supported by the results?

Yes

Are sufficient details of methods and analysis provided to allow replication by others?

Yes

Reviewer Expertise:

Pulmonology diseases

I confirm that I have read this submission and believe that I have an appropriate level of expertise to confirm that it is of an acceptable scientific standard, however I have significant reservations, as outlined above.

## References

[1] : Combined use of rheology and portable low-field NMR in cystic fibrosis patients. *Respir Med* .2021;189: 10.1016/j.rmed.2021.106623 106623 10.1016/j.rmed.2021.106623 34624628

[2] : Prevalence and Impact of Rheumatologic Pain in Cystic Fibrosis Adult Patients. *Front Med (Lausanne)* .2021;8: 10.3389/fmed.2021.804892 804892 10.3389/fmed.2021.804892 35211488PMC8861186

# Reviewer report: Reviewer response for version 1

**DOI:** 10.3310/nihropenres.14515.r29758

**Published:** 2023-08-10

**Authors:** Dana Albon

**Affiliations:** 1 UVA, Virginia, USA

This is a very well written manuscript. It describes the results of a survey aiming to investigate the presence of gastrointestinal symptoms in teenagers and adults with CF post modulator therapy use. Most people with CF answering the survey were on ETI. 

 I recommend minor revisions to the manuscript. 

 Under results, I recommend for the authors to review the numbers again; they do not add and from line to line they differ. Example: for PwCF 89/110 were prescribed a modulator, however the ETI was reported in 73/84. If 89 were prescribed a modulator, the reported ETI use would need to be 73/89 and not 84. There are several discrepancies like this. Please also review the demographics table; same discrepancies are seen in the table; PwCF are listed at 88 and not 89. Similar discrepancies exist throughout the results.

Is the work clearly and accurately presented and does it cite the current literature?

Yes

If applicable, is the statistical analysis and its interpretation appropriate?

Not applicable

Are all the source data underlying the results available to ensure full reproducibility?

Yes

Is the study design appropriate and is the work technically sound?

Yes

Are the conclusions drawn adequately supported by the results?

Yes

Are sufficient details of methods and analysis provided to allow replication by others?

Yes

Reviewer Expertise:

I am a Pulmonary Critical Care physician who has been taking care of CF patients for more than 10 years.

I confirm that I have read this submission and believe that I have an appropriate level of expertise to confirm that it is of an acceptable scientific standard, however I have significant reservations, as outlined above.

# Reviewer report: Reviewer response for version 1

**DOI:** 10.3310/nihropenres.14515.r29985

**Published:** 2023-08-10

**Authors:** Borowitz Drucy

**Affiliations:** 1 University at Buffalo, Buffalo, New York, USA

This manuscript addresses a timely topic and its strength is that it is grounded in the lived experience of pwCF. My specific comments are as follows:

**Abstract:** I'd delete focusing on changes made in medications in the abstract and add some of the most relevant findings e.g. the improvement in appetite, and the discrepancy between the perception of 38% of pwCF that stomach pain improved whereas 62% of HCPs thought it was improved. (See my comments below)

**Plain language summary:** Well-written, although I might conclude that based on this study's results, future research into the effects of CFTR modulators needs to focus on GI and QoL issues.

**Introduction:** No changes needed

**Methods:** Well-described

**Results**:

1. I cannot determine how many respondents were not taking modulators. After the bold section titled "Symptoms Experienced", 19 is reported as the denominator. (Note that there seems to be a word missing at the start of this sentence). One sections states, " *Of those not prescribed a modulator (n=20)...*", though I suspect this means those not taking a modulator since 4 were reported as having discontinued treatment, therefore they must have had modulators prescribed at some point. The explanation for Figure 2c says 38 subjects were not on a modulator. This discrepancy needs to be resolved.
2. Figure 2c should include the n for each column so that the reader doesn't have to go back to Figure 1 (especially since the number of people not on modulators is much lower than the number reporting in 2018). 3) The section on Diet and Medication changes is interesting, especially the reported changes in diet. However, I would delete the paragraph that start, " *For pwCF taking a CFTR modulator and HCPs, the symptoms of constipation...*" This doesn't add anything and it is unclear how many pwCF responded/ how many HCPs responded in free text.
  The number is likely to be too small for this to have any generalizable information. I would also entirely delete the section on HCPs and PERT. The lack of objective measures or correlation with specific patients is confusing. It is more important to report that some pwCF made changes to their PERT doses without the advice of HCPs (your item 2 in this section; the last paragraph). This may be useful to HCPs who may be unaware that pwCF are doing this and can open future conversations.

**Discussion**:

1. Some of the key findings are not discussed. e.g. Figure 1 shows that DIOS fell from 39% in 2018 to 29% in 2022. Although meconium ileus fell similarly it is unlikely that this neonatal condition changed in that short a period of time. However, the decline in DIOS, even if it is an artifact, deserves some discussion especially since this is a significant GI complication
2. Figure 2 indicates that lack of appetite and stomach cramps appears to be less in people on modulators. Again, the numbers in the groups are not comparable, but why do the authors speculate this might be?
3. Based on Figure 3, 38% of pwCF thought stomach pain had improved on modulators vs. 62% of HCPs thought stomach pain had improved. The authors should discuss this discrepancy and how it might stimulate future patient-HCP conversations.
4. The limitations paragraph should note that it is difficult to compare those on and off modulators because the size of the groups is not the same and the data may be skewed by the small number of subjects not on modulators.

**Figures**: Figure 3: I might title 3b " *How pwCF describe their symptoms to HCPs*". Figure 4 is unnecessary. The text does a better job of describing the themes.

Is the work clearly and accurately presented and does it cite the current literature?

Yes

If applicable, is the statistical analysis and its interpretation appropriate?

Partly

Are all the source data underlying the results available to ensure full reproducibility?

Yes

Is the study design appropriate and is the work technically sound?

Yes

Are the conclusions drawn adequately supported by the results?

Partly

Are sufficient details of methods and analysis provided to allow replication by others?

Yes

Reviewer Expertise:

CF clinical trials; PERT trials; evaluation of GI symptoms; methods to include the lived experience of pwCF

I confirm that I have read this submission and believe that I have an appropriate level of expertise to confirm that it is of an acceptable scientific standard, however I have significant reservations, as outlined above.

# Reviewer report: Reviewer response for version 1

**DOI:** 10.3310/nihropenres.14515.r29764

**Published:** 2023-08-10

**Authors:** Mette Frahm Olsen

**Affiliations:** 1 University of Copenhagen, Copenhagen, Denmark

The manuscript “A grumbling concern: an international survey of gastrointestinal symptoms in cystic fibrosis in the modulator era” describes the findings of a study investigating GI symptoms from the lived experience and its impact of quality of life. The study finds that GI symptoms remain a problem in CF and highlights the need for a better understanding of the pathophysiology of GI symptoms.

 Relief of GI symptoms is a highly prioritised topic among those living with CF, their families/friends and CF professionals. Authors should be commended for involving the CF community in this study as members of a study steering group and as a co-author of the paper.

General comments

 It seems a bit of a stretch to call this “an international survey” as it was only in English and mainly promoted on UK platforms. The large majority of respondents (85%) were from the UK and the rest mainly from North America. It leaves about 10 respondents from other European countries. “<5% rest of world” is mentioned but other categories sum to 100%. I would suggest toning down that it was international (from title etc). If it was a main aim, the survey should have been planned differently to better represent the international CF community.

 No direct comparisons were possible, but data suggest that GI symptoms persist after introduction of CFTRm. However, a main issue with the study is selection bias as people self-select to answer a survey about GI symptoms, i.e. issues are likely to be overrepresented and potential improvement could be overlooked. Selection bias is mentioned in study limitations, but not the risk of not detection improvements, e.g. if those with resolved symptoms are less likely to answer the survey.

Abstract

 Changes in dietary intake and medication are reported. It is later mentioned that these are with the intention of managing GI symptoms. Please add this information to the abstract.

Introduction

 You argue that “ *These results will inform the development of a CF-specific gastrointestinal patient reported outcome measure for people with CF that can be used in clinical trials.*” But as mentioned just above, several CF-specific tools exist. Please clarify how the new tool will be different from the currently available tools? (e.g. Mainz et al. CFAbd score, Quittner et al. GI symptom tracker), and why there is a need for more PROMs?

Results

 The results are partly based on qualitative data from free-text fields in survey. Please indicate how much data this is based on, e.g. how many respondents used this option and how much text did they provide?

 There are 167 participants in the study, but the denominator is much lower for many variables. Was modulator treatment only known for 110? And only 103 provided responses for GI symptoms? Please clarify and mention in study limitations if this is the case.

 Fig 2. Symptoms by modulator status. Based on 2022 data? Or also 2018 data? Not clear. If only 2022, no modulator data is based on 20 participants? Number of participants should be included in the figure or figure legend.

 Fig 3. HCP seem to overestimate the effects of modulators on GI symptoms. But this could be due to selection bias among CF patients > HCP.

Discussion

 It is not the same respondents or questions in the 2018 vs 2022 surveys, which makes a comparison difficult. Authors do mention this limitation, but I think it would be reasonable to add that longitudinal studies are needed to assess prevalence and changes in GI symptoms in CF.

Is the work clearly and accurately presented and does it cite the current literature?

Yes

If applicable, is the statistical analysis and its interpretation appropriate?

Not applicable

Are all the source data underlying the results available to ensure full reproducibility?

Yes

Is the study design appropriate and is the work technically sound?

Partly

Are the conclusions drawn adequately supported by the results?

Partly

Are sufficient details of methods and analysis provided to allow replication by others?

Yes

Reviewer Expertise:

Cystic fibrosis; public health; epidemiology

I confirm that I have read this submission and believe that I have an appropriate level of expertise to confirm that it is of an acceptable scientific standard, however I have significant reservations, as outlined above.

# Reviewer report: Reviewer response for version 1

**DOI:** 10.3310/nihropenres.14515.r29317

**Published:** 2023-07-12

**Authors:** Dhiren Patel

**Affiliations:** 1 Saint Louis University, St. Louis, Missouri, USA

I congratulate authors to conduct this survey. This is well needed guidance from patients and CF care providers perspective to CF researcher and shows that ETI may have fixed some issues and unfortunately Gastrointestinal symptom is not one of them.

 Few comments for authors:

 Although it is named as international survey, the majority of responders were from UK. This could have been an issue with the distribution. I certainly have not seen this survey making its way to me in USA and I am part of the DIGEST group in USA. Having said that, this has been done in USA and authors cite that reference in the manuscript. I don't think its a big issue but I would suggest to specify this as a limitation.

 Reviewer Comments: A grumbling concern

**Abstract:**

1. Consider replacing “rumbling stomach noises” with borborygmi
2. “ *loose motions (modulator) and bloating (no modulator)*" – does this mean loose motion is the most commonly reported in patients on modulator etc?

**Page 3:**

1. Correct spelling of “licencing”
2. “ *prior to the licensing of the newest and most widely used modulator ETI*” – no comma needed
3. Resources in [ ] are great, but seem out of place as just a link here. Perhaps consider keeping them as a reference and then putting together a list of the hyperlinks at the end.
4. 5 ^th^ paragraph, first sentence, consider condensing this sentence
       
**- **5 ^th^ paragraph, last sentence, add a source
5. 6 ^th^ paragraph: consider changing less well to not as well
6. 7 ^th^ paragraph: Spell our JLA the first time you use it
7. Keep references consistent

**Page 4:**

1. 1 ^st^ paragraph: PSP?
2. 2 ^nd^ paragraph: consider changing patient community to CF community
   
** -** At the end of this paragraph, the reference style is not consistent with other references

**Page 5:**

1. First paragraph – your point regarding artificially increasing words is a good point
2. Table 1: what does “Other” vs “Unknown” entail?

**Page 6:**

1. Figure 1: clarify what is in other vs none of the above

**Page 7:**

1. Figure 3 – interesting discrepancy between HCP vs PwCF reports of changes in symptoms

**Page 8:**

1. Paragraph 6 – last line – what sort of changes to advice did they give? 

Is the work clearly and accurately presented and does it cite the current literature?

Yes

If applicable, is the statistical analysis and its interpretation appropriate?

Yes

Are all the source data underlying the results available to ensure full reproducibility?

No source data required

Is the study design appropriate and is the work technically sound?

Yes

Are the conclusions drawn adequately supported by the results?

Yes

Are sufficient details of methods and analysis provided to allow replication by others?

Yes

Reviewer Expertise:

Pediatric Gastroenterology, Hepatology and Nutrition, Cystic Fibrosis, Gastrointestinal Motility

I confirm that I have read this submission and believe that I have an appropriate level of expertise to confirm that it is of an acceptable scientific standard.
